# Discovery of two novel mutations, R52H and N868D, in the voltage-gated sodium channel of *Aedes aegypti* associated with pyrethroid resistance

**DOI:** 10.1371/journal.pntd.0014393

**Published:** 2026-05-26

**Authors:** Han-Hsuan Chung, Hwa-Jen Teng, Shiu-Ling Chen

**Affiliations:** Center for Diagnostics and Vaccine Development, Centers for Disease Control, Ministry of Health and Welfare, Taipei, Taiwan; Institut Pasteur du Cambodge, CAMBODIA

## Abstract

*Aedes aegypti* is the primary vector of arboviruses, including the dengue virus. Insecticide-based vector control remains a key strategy for reducing mosquito populations and disrupting disease transmission; however, its effectiveness is increasingly challenged by resistance. Identifying resistance mechanisms is crucial for monitoring resistance trends and informing policy decisions for vector control and disease prevention. We aimed to investigate the voltage-gated sodium channel *(vgsc)* mutations in pyrethroid-resistant *Ae. aegypti* strains. In this study, we established a cypermethrin-resistant *Ae. aegypti* strain from field-collected mosquitoes in 2016 and performed whole-genome sequencing to identify resistance-associated *vgsc* mutations. Additionally, we genotyped field *Ae. aegypti* collected in 2024 to assess the variation and prevalence of resistance-related mutations. Our findings confirmed that R52H and N868D are associated with resistance, with R52H co-occurring with S989P+V1016G and N868D co-occurring with F1534C. The widespread presence of R52H and N868D, along with their co-circulation with other resistance-associated mutations in the field population, suggests that these mutations have been maintained under intense selection pressure. Furthermore, we identified 12 haplotypes and suggested a potential evolutionary trajectory based on co-occurrence patterns of mutation events. The emergence and persistence of novel *vgsc* mutations in field populations highlight the ongoing expansion of insecticide resistance in Taiwan and globally, which threatens the efforts for disease vector control. Understanding the functional impact of these mutations is essential for comprehensive monitoring and evaluating pyrethroid resistance dynamics. Systematic surveillance program is required to track resistance trends and guide evidence-based vector control strategies that ensure effective disease prevention and public health protection.

## Author summary

Long-term chemical interventions exert strong selection pressure, driving the emergence of resistance-associated mutations and ultimately compromising the effectiveness of vector control strategies. In this study, we identified two *vgsc* mutations, R52H and N868D, that are associated with pyrethroid resistance, based on both a cypermethrin-resistant laboratory strain and field surveillance data. Our findings highlight the growing threat of insecticide resistance in *Aedes aegypti* populations in Taiwan and globally, and underscore the need for further investigation into the functional impacts of these mutations.

## Introduction

The yellow fever mosquito, *Aedes aegypti*, has become one of the most significant vectors of arboviruses globally, spreading pathogens such as dengue, Zika, chikungunya, and yellow fever viruses. Among them, the dengue virus is the most prevalent pathogen affecting residents in tropical and subtropical regions [1]. According to an estimation, approximately 390 million people are infected with dengue annually, posing noticeable health and economic challenges worldwide [2]. In Taiwan, dengue occurs annually, and indigenous cases have ranged from tens to tens of thousands of dengue fever cases in the past 2 decades (https://www.cdc.gov.tw/). In 2015, a record was established with 43,419 indigenous cases, which declined in subsequent years. However, the dengue outbreak re-emerged in 2023, with 26,429 indigenous cases. Because there is no specific antiviral treatment or cost-effective vaccine for dengue [3], disease prevention primarily depends on mosquito control measures, including environmental management and space spraying of insecticides in areas surrounding dengue cases [4]. However, the repeated use of insecticides with the same mode of action has led to the development of resistance in mosquito populations [5]. In Taiwan, pyrethroids are the primary insecticide used for *Aedes* mosquito control because of their high toxicity toward pests and low adverse effects on mammals [6]; however, *Ae. aegypti* larvae and adults have exhibited resistance to pyrethroid [7–9].

One of the mechanisms leading to insecticide resistance is knockdown resistance [5]. Mutation in voltage-gated sodium channel (*vgsc*), which is the target site of pyrethroids, reduces the binding affinity between pyrethroid and target protein and subsequently results in resistance [10]. In *Ae. aegypti*, several *vgsc* mutations have been reported to be associated with pyrethroid resistance, including V253F, V410L, G923V, L982W, S989P, A1007G, I1011V/M, V1016G/I, T1520I, F1534C, and D1763Y [11–18]. In Taiwan, nine haplotypes comprising S989P, V1016G, T1520I, F1534C, and D1763Y and two intron polymorphisms between exons 20 and 21 have been observed in the field population [19,20]. Of them, V1016G+D1763Y and S989P+V1016G have been demonstrated to be associated with type I and type II pyrethroid resistance, respectively [9,19,21]. An 8-year surveillance (2016–2023) of resistance-associated *vgsc* mutations in *Ae. aegypti* in Taiwan monitored the trend of resistance over time and showed that the specific site-unmutated wild type has been absent since 2020. Instead, resistance-associated *vgsc* mutations were predominant in the field population, indicating an increasing resistance status of field *Ae. aegypti* populations [20].

With the availability of high-resolution reference genome sequences of insects with public health and agricultural importance and advancements in whole-genome sequencing (WGS) technologies, large-scale genome surveillance has been performed, and novel resistance-associated mutations have been discovered [22–26]. For example, V402L+I1527T in *vgsc* of *Anopheles coluzzii* was identified, and its role in pyrethroid resistance was comparable to that of the well-proven L995F [27]. In addition, I4790K in the ryanodine receptor of *Plutella xylostella*, associated with diamide resistance, was first discovered in Japan [23]. Later, studies with samples from various countries indicated that this mutation is potent for diamide resistance [28,29]. In *Ae. aegypti,* several novel mutations have been identified to be associated with insecticide resistance using the WGS platform [11,30,31]. In Taiwan, all monitoring efforts for *vgsc* resistance mutations focused exclusively on previously reported or functionally characterized sites [9,19–21]. However, identifying previously unrecognized resistance-associated mutations helps to comprehensively evaluate the resistance status and trend. In this study, we used the WGS technique to investigate the *vgsc* gene of cypermethrin-selected *Ae. aegypti* strain. We aimed to understand whether additional mutations contribute to pyrethroid resistance and to discuss the relationship between *vgsc* mutations and their resistance phenotype.

## Materials and methods

### Establishment of cypermethrin-selected *Aedes aegypti* strain

**Fig 1:**
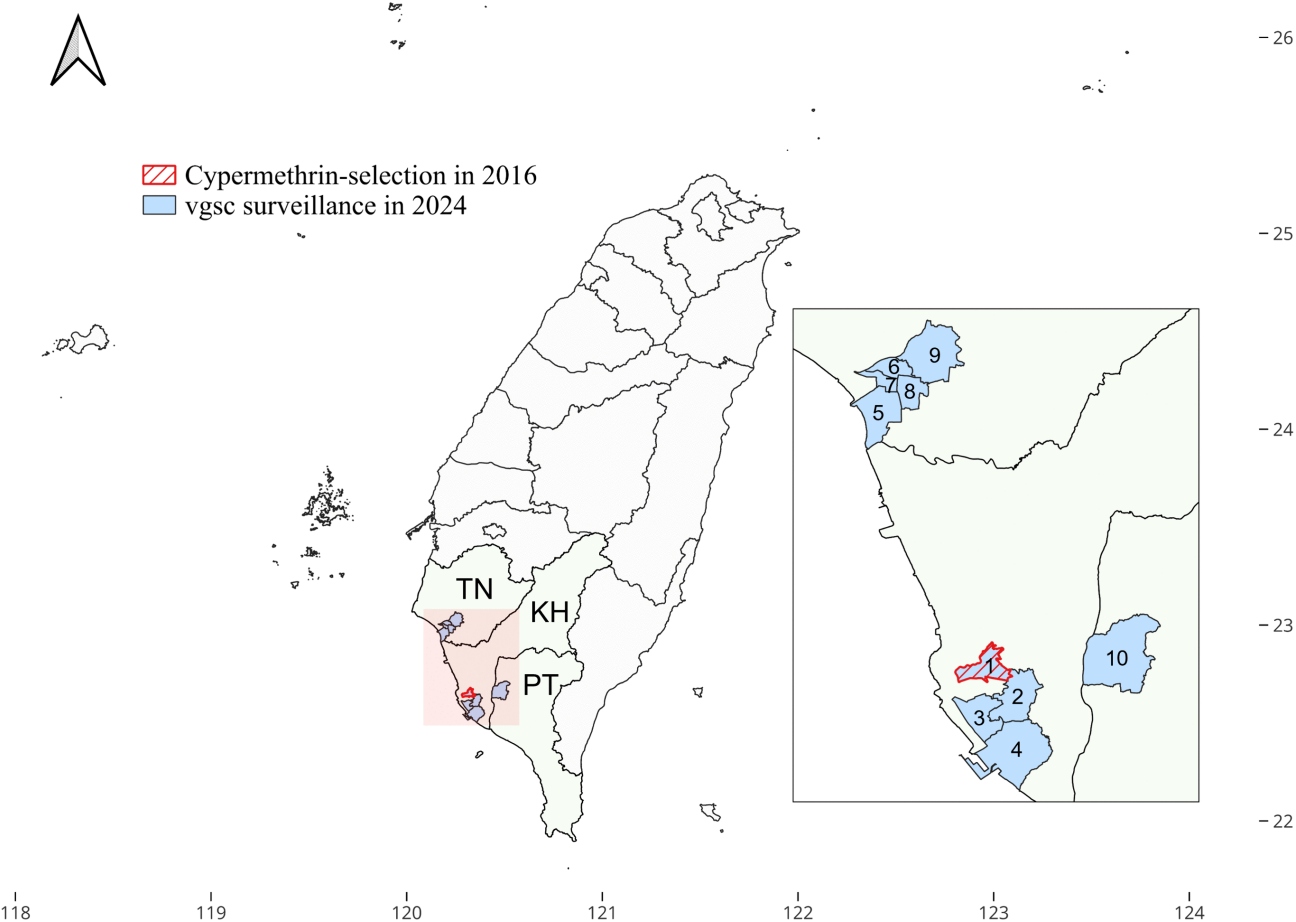
Locality of *Aedes aegypti* sampling. The district where *Ae. aegypti* was collected for cypermethrin selection in 2016 is labeled in red. Mosquitoes for *vgsc* mutation surveillance in 2024 was collected by the National Mosquito-Borne Diseases Control Research Center, National Health Research Institutes in 10 districts (labeled in blue) of southern Taiwan, including Sanmin (1), Fengshan (2), Qianzhen (3), Xiaogang (4) of Kaohsiung City (KH), South (5), North (6), West Central (7), East (8), Yongkang (9) of Tainan City (TN), and Pingtung (10) of Pingtung County (PT). The map was created using QGIS 3.32.2 (https://qgis.org). The base layer of the map with CC BY 4.0 license (https://data.gov.tw/licenses) was downloaded from Government Open Data established by National Development Council, Taiwan (https://data.gov.tw/dataset/7442).

Morphologically confirmed immature *Ae. aegypti* were collected from standing water containers in the Sanmin district of Kaohsiung city in 2016 because the district is a dengue hotspot area (1 in Fig 1). The larvae were reared to adulthood in the insectary without insecticide exposure, and their offspring (G0) were used for insecticide selection, following a previously reported procedure [19]. Briefly, the field-collected larvae were reared in a plastic pan containing a 3:1 mixture of pig liver powder and yeast extract. Adult mosquitoes were kept in a BugDorm screen cage (30 × 30 × 30 cm; MegaView Science, Taichung, Taiwan) under a 10:14 light:dark cycle at 20–30 °C and 70 ± 10% relative humidity. A 10% sucrose solution was used as the energy source for adult mosquitoes. The adults were reared and fed specific-pathogen-free pig blood (Taitung District, Livestock Research Institute, Ministry of Agriculture, Taiwan) to oviposit the next generation (G0) for subsequent pyrethroid selection. A commercial insecticide with 5% w/w cypermethrin (Cyper-5 from TYENG LONG Incorporation) was used for selection because it is commonly used by local government for vector control. To determine the median lethal concentration (LC_50_) used for selection, a preliminary test was performed by exposing mosquitoes to various concentrations of commercial cypermethrin, according to a small-scale study of World Health Organization guidelines [32,33]. Briefly, for each concentration, 110 3-day-old to 7-day-old adult females were separated into 11 net cages (10 adults per net cage). Ten cages were randomly hung in an exposure room (approximately 30 m^3^), while the remaining cage served as the unexposed control. An air-atomizing nozzle (SU1A; Spraying Systems Co., U.S.) sprayed varied concentrations of cypermethrin diluted with distilled water (S1 Table). After 30 min of exposure, the mosquitoes were transferred into a collection chamber (BioQuip Products Inc., Rancho Dominguez, CA, USA) and supplied with a 10% sucrose solution on cotton pads. The mosquitoes were subsequently placed in an incubator at 27 ± 2 °C and 75 ± 10% relative humidity. The knockdown rate and mortality (S1 Table) were recorded for 30 min and 24 h post-exposure, respectively, according to the criteria described in the World Health Organization guidelines [34]. The LC_50_ was determined and used to select 250 mosquitoes (25 adults per net cage for 10 net cages) of both sexes from the same generation using the aforementioned procedure. Similarly, one net cage (25 adults) serves as the unexposed control. After selection, live mosquitoes were collected, reared, and fed specific-pathogen-free pig blood to oviposit the next generation (G1). The G1 mosquitoes were used to perform the selection experiment to obtain the next generation. Sequential selection was performed up to G5. Mosquitoes were randomly picked from each generation before cypermethrin exposure and stored at –80 °C for *vgsc* genotyping.

### Library preparation, sequencing, and variant analysis using WGS

Ten female mosquitoes from G0, G3, and G6 cypermethrin-selected mosquitoes were pooled for DNA extraction using Qiagen QIAamp DNA purification kit (cat. no. 51306; Qiagen, Germany) and subjected to WGS (Welgene Biotech Co., Ltd., Taipei, Taiwan). Briefly, 3 µg of total DNA from each generation was sonicated using Covaris M220 sonicator to sizes ranging from 300 bp to 400 bp. The DNA size was assessed using a TapeStation D1000 chip (Agilent Technologies). Then, 1 µg of sonicated DNA was end-repaired, A-tailed, and adaptor-ligated, following the Illumina’s TruSeq DNA preparation protocol. The constructed DNA library was sequenced on the Illumina Sequencer using the 150 bp paired-end protocol. The 150 bp paired-end raw reads were obtained after adaptor removal and quality trimming using Trimmomatic [35]. The trimmed reads were aligned using Burrows–Wheeler Aligner [36] against the reference genome sequence of *Ae. aegypti* Liverpool AGWG strain (AaegL5, downloaded from the VectorBase database). To facilitate variant analysis further, variant calling and annotation were performed using LoFreq [37] and SnpEff [38].

### Field collection of *Ae. aegypti*

*Ae. aegypti* samples from southern Taiwan in 2024 were provided by the National Mosquito-Borne Diseases Control Research Center, National Health Research Institute. These samples were collected from 10 districts at high risk of dengue in southern Taiwan, including five districts of Tainan City (West Central District, South District, North District, East District, and Yongkang District), four districts of Kaohsiung City (Sanmin District, Xiaogang District, Qianzhen District, and Fengshan District), and Pingtung City in Pingtung County (Fig 1). Males were selected, preserved in absolute ethanol, and stored at –80 °C until *vgsc* genotyping. Additionally, live and dead samples of field G0 *Ae. aegypti* from southern Taiwan in 2016 after cypermethrin exposure were obtained from a previous study [21].

### *vgsc* genotyping

Genomic DNA was extracted manually using Qiagen QIAamp DNA purification kit in 2016 (cat. no. 51306; Qiagen, Germany), and automatically using Maelstrom 4800 (Taiwan Advanced Nanotech Inc., Taiwan) with TANBead Tissue Total DNA Auto Plate (cat. no. M6T2A46; Taiwan Advanced Nanotech Inc., Taiwan) in 2024. For cypermethrin-selected strain, 20 male mosquitoes were randomly chosen from G0–G6. For field-collected mosquitoes in 2024, 10 male mosquitoes from each of 10 districts were genotyped. For G0 live and dead mosquitoes after cypermethrin exposure in 2016 [21], we prioritized the analysis of the N868D mutation due to its high prevalence and significant dose-response trend observed during cypermethrin selection. A total of 87 and 29 females *Ae. aegypti* from Kaohsiung (live, n = 28; dead, n = 59) and Tainan (live, n = 10; dead, n = 19) cities, respectively, were genotyped. Conversely, R52H, D126N, P162H, and A532E were evaluated using a representative subset to assess their baseline presence. This subset comprised 29 and 13 females *Ae. aegypti* from Kaohsiung (live, n = 18; dead, n = 11) and Tainan (live, n = 8; dead, n = 5) cities, respectively. Briefly, each mosquito was individually homogenized with a 3-mm glass bead in a 1.5 mL microcentrifuge tube for 3 min at a frequency of 30/s using a TissueLyser (Qiagen, Germany). The homogenized sample was then processed according to the manufacturer’s instructions, and the genomic DNA was eluted with the elution buffer. The *vgsc* gene was genotyped as described previously [19,39]. Partial DNA fragments of *vgsc* containing R52, D126, P162, A532, N868, S989, V1016, T1520, F1534, and D1763 (numbered based on the *Musca domestica vgsc*: GenBank: AAB47604) were amplified using specific polymerase chain reaction primers (S2 Table) [39] and a thermocycler (Biometra T3000, Germany). The amplification products were separated using electrophoresis on a 1.5% agarose gel and visualized on an ultraviolet light box following DNA staining (RedSafe, cat. No 21141; iNtRON BIOTECHNOLOGY, Korea). Amplicons were sent for direct sequencing (Genomics, Taiwan) using the designated sequencing primers (S2 Table) [39]. The *vgsc* genotypes of each allele were aligned and analyzed using the GeneStudio software (http://genestudio.com/). The haplotypes were determined as previously described [20]. The co-occurrence of each *vgsc* mutation was analyzed according to the *vgsc* genotypes of field-collected *Ae. aegypti* 2024.

### Statistical analysis

All statistical analyses were performed using R (version 4.4.2; The R Foundation for Statistical Computing, Vienna, Austria) [40]. Statistical analyses were performed as follows: (1) Fisher’s exact test was used to compare zygosity distribution between live and dead mosquitoes from Tainan and Kaohsiung in 2016; (2) Pearson’s correlation coefficient was used to analyze relationships between: (i) cypermethrin LC_50_ and mosquito generation, (ii) allele frequency of mutations and cypermethrin LC_50_, (iii) haplotype percentages and cypermethrin LC_50_ in the cypermethrin-selected strain, and (iv) co-occurrence of mutation sites in field-collected *Ae. aegypti* from 2024.

## Results

During the establishment of cypermethrin-selected *Ae. aegypti* strains, the unexposed control groups in each treatment exhibited 100% survivorship. Compared with the G0 population, the resistance ratio significantly increased over the generations, and the resistance ratio of the G5 population was 7.61-fold higher (Table 1). The knockdown rate and mortality of G0–G5 in the cypermethrin selection experiment are shown in S3 Table.

**Table 1:** LC_50_ of cypermethrin and resistance ratio in G0–G5 *Ae. aegypti.*

Generation	LC_50_ (mg/mL)	RR	*r*	*p* value
G0	0.0571	1	0.88	0.019
G1	0.0674	1.18		
G2	0.1656	2.90		
G3	0.1695	2.97		
G4	0.1887	3.30		
G5	0.4348	7.61		

Resistance ratio (RR): LC_50_ of G1–G5/LC_50_ of G0. *r* = correlation coefficient between generation and LC_50._

**Fig 2:**
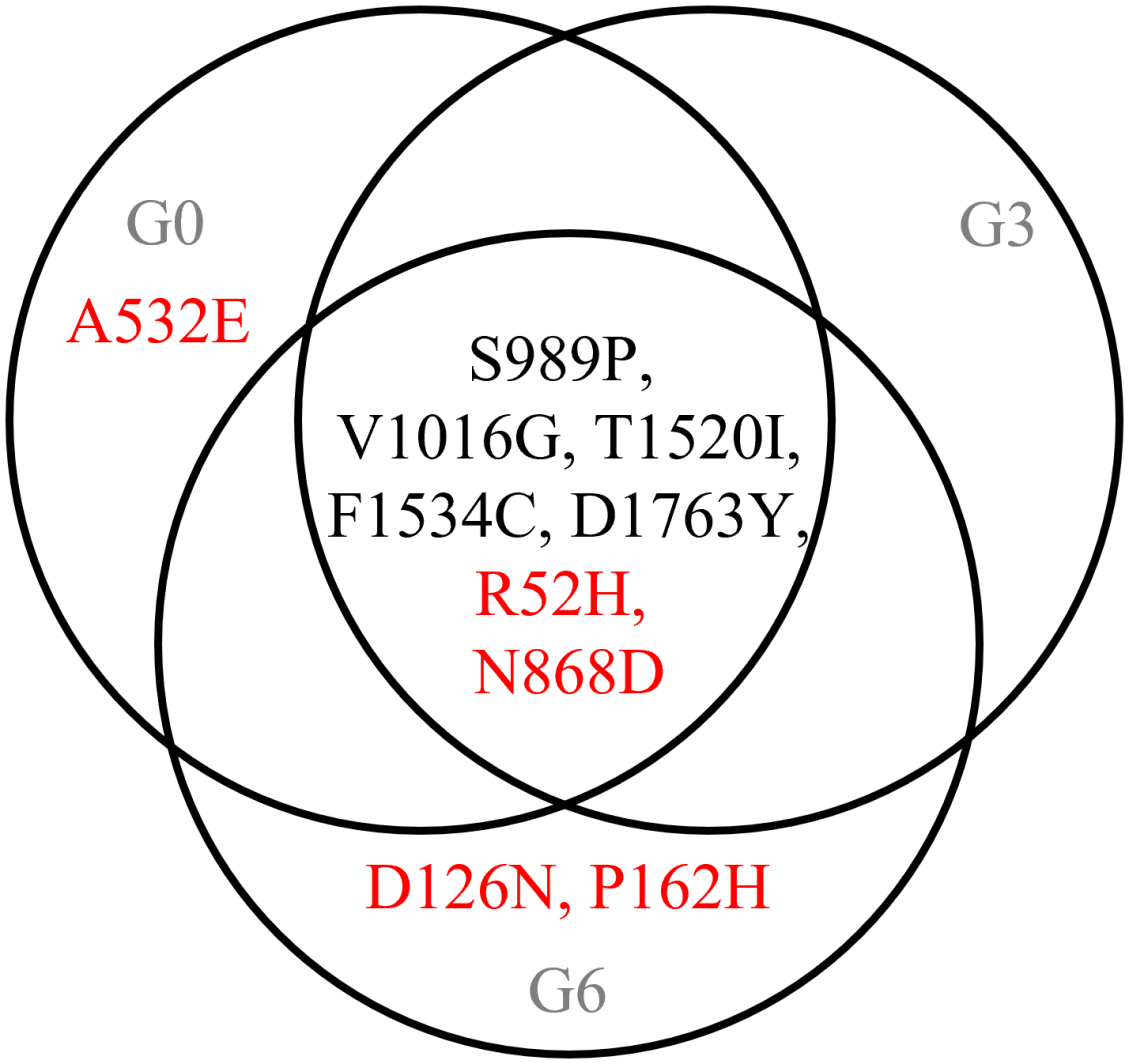
Non-synonymous *vgsc* mutations in cypermethrin-selected G0, G3, and G6 *Aedes aegypti* suggested by whole-genome sequencing analysis are shown in the Venn diagram. The mutations previously reported in *Ae. aegypti* in Taiwan are labeled in black. The mutations detected using whole-genome sequencing in this study are labeled in red.

The DNA of G0, G3, and G6 was subjected to WGS. The sequencing data for this study are available in the NCBI Sequence Read Archive (SRA) under the BioProject accession number PRJNA1370646. The summary of sequencing results is presented in S4 Table. The total sequence of each processed generation ranged from 122 million to 533 million. The sequence qualities were higher than the Q30 level, with per sequence quality score of 36. In addition to the *vgsc* mutations that have been reported in Taiwan (S989P, V1016G, T1520I, F1534C, and D1763Y), we further detected five non-synonymous mutations, including R52H (nucleotide codon: CGC to CAC, arginine at site 52 was replaced by histidine) and N868D (AAC to GAC, asparagine at site 868 was replaced by aspartic acid) in G0, G3, and G6 samples; A532E (GCA to GAA, alanine at site 532 was replaced by glutamic acid) in G0 sample; and D126N (GAT to AAT, aspartic acid at site 126 was replaced by asparagine) and P162H (CCT to CAT, proline at site 162 was replaced by histidine) in G6 sample (Fig 2). However, we did not detect V253F, V410L, G923V, L982W, A1007G, and I1011V/M in G0, G3, and G6 cypermethrin-selected mosquitoes.

**Fig 3:**
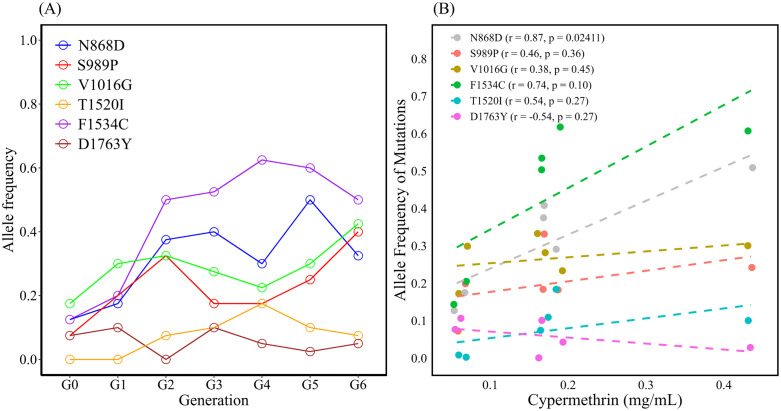
Variation in allele frequencies of N868D, S989P, V1016G, T1520I, F1534C, and D1763Y (A) and the relationship between these mutations and the LC_50_ of cypermethrin (B) in sequential cypermethrin-selected *Aedes aegypti.* The correlation coefficient (r) and p-value (p) for each mutation are shown in parentheses. The linear regression line is represented by the dashed line.

We verified the mutations identified via WGS by genotyping individual mosquitoes from G0, G3, and G6. The frequency of N868D (chromatogram is shown in S1A Fig) in G0, G3, and G6 was 0.125, 0.4, and 0.325, respectively, suggesting an increasing trend. R52H (S1B Fig) was detected only in G6 at a frequency of 0.15. We detected D126N and P162H in the later phase of selection. The frequency of D126N in G3 and G6 was 0.025, and the frequency of P162H in G6 was 0.1, indicating low mutation frequency. The allele frequencies of A532E in G0, G3, and G6 were 0.1, 0.025, and 0.025, respectively, suggesting a decreasing trend (Table 2). Because of the frequent occurrence of N868D in G0, G3, and G6, this mutation was analyzed in other generations. In addition, the mutations reported in previous studies were genotyped for comparison. The mutation frequency of N868D increased over the generations, peaking at 0.5 in G5, and then decreased to 0.325 in G6 (Fig 3A and S5 Table). We also observed that the frequency of F1534C, the predominant mutation in the G0–G6 population, increased to 0.625 in G4 and then decreased to 0.5 in G6. The trends of S989P and V1016G were similar, with an increasing trend over the generations and peak of 0.4 and 0.425, respectively, in G6. The trend of T1520I was similar to that of F1534C but with a low frequency and peak in G4 (0.175). The frequency of D1763Y was generally below 0.1. We also analyzed the correlation between the frequency of each mutation and the LC_50_ of cypermethrin in G0–G5. Although all mutation frequencies were positively correlated with LC_50_, except for D1763Y mutation frequency, only N868D mutation frequency showed a statistically significant difference (Fig 3B).

**Table 2:** Mutation frequency of R52H, D126N, P162H, A532E, and N868D in G0, G3, and G6 cypermethrin-selected *Aedes aegypti.*

Generation	Number^*^	R52H	D126N	P162H	A532E	N868D
G0	20	0	0	0	0.1	0.125
G3	20	0	0.025	0	0.025	0.4
G6	20	0.15	0.025	0.1	0.025	0.325

* indicates the number of mosquitoes tested in each generation.

To validate the potential resistance-associated mutations suggested in the laboratory-selected strain, field-collected *Ae. aegypti* samples (2016) that were previously exposed to commercial cypermethrin and categorized as live or dead were genotyped [21]. The allele frequency of N868D in live mosquitoes was significantly higher than that in dead mosquitoes (Tainan, 0.250 vs. 0.184; Kaohsiung, 0.179 vs. 0.025; Overall, 0.197 vs. 0.067) (Table 3). The allele frequency of R52H in live mosquitoes was higher than that in dead mosquitoes (0.077 vs. 0). However, no significant difference was observed between the groups. In contrast, all the field *Ae. aegypti* were wild type for D126N, P162H, and A532E (S6 Table).

**Table 3:** Allele frequency of N868D in live and dead field-collected *Ae. aegypti* after cypermethrin exposure.

Mutation		Phenotype	Number*	N/N	N/D	D/D	Allele frequency	*p* value
N868D	Tainan	Alive	10	5	5	0	0.250	0.015
		Dead	19	15	1	3	0.184	
	Kaohsiung	Alive	28	19	8	1	0.179	0.001
		Dead	59	56	3	0	0.025	
	Total	Alive	38	24	13	1	0.197	0.000
		Dead	78	71	4	3	0.067	

* indicates the number of mosquitoes tested in each generation.

The N/N, N/D, and D/D indicate the wild type, heterozygous mutation, and homozygous mutation of N868D, respectively.

To understand the role of N868D in resistance further and confirm whether this mutation is suitable for preservation in the field, we genotyped *Ae. aegypti* strains collected from southern Taiwan in 2024 (Table 4). The average mutation frequency of N868D was 0.305, with a range of 0.05 to 0.85. The frequencies of S989P, V1016G, T1520I, F1534C, and D1763Y were 0.55, 0.635, 0.055, 0.375, and 0.115, respectively. We did not detect D126N and A532E in the field population of *Ae. aegypti*. We only detected P162H with a low average frequency of 0.01 (only in the Fengshan district). Surprisingly, we observed R52H in all districts with an average frequency of 0.265 (range, 0.05 to 0.65), although only a low frequency of this mutation was observed in cypermethrin-selected *Ae. aegypti* (Table 2).

**Table 4:** Allele frequency of *vgsc* mutations in the field population of *Aedes aegypti* in 2024.

City	District	Number*	R52H	D126N	P162H	A532E	N868D	S989P	V1016G	T1520I	F1534C	D1763Y
Tainan	Yongkang	10	0.65	0	0	0	0.05	0.85	0.9	0.05	0.1	0.05
	East	10	0.05	0	0	0	0.6	0.2	0.4	0	0.6	0.2
	South	10	0.1	0	0	0	0.85	0.15	0.15	0	0.85	0.05
	West Central	10	0.25	0	0	0	0.45	0.35	0.55	0.05	0.45	0.2
	North	10	0.35	0	0	0	0.05	0.8	0.95	0	0.05	0.25
Kaohsiung	Fengshan	10	0.45	0	0.1	0	0.05	0.75	0.8	0.05	0.2	0.05
	Sanmin	10	0.1	0	0	0	0.25	0.75	0.75	0	0.25	0.05
	Qianzhen	10	0.2	0	0	0	0.2	0.65	0.65	0.15	0.45	0.05
	Xiaogang	10	0.25	0	0	0	0.15	0.55	0.6	0.25	0.4	0.1
Pingtung	Pingtung	10	0.25	0	0	0	0.4	0.45	0.6	0	0.4	0.15
Mean			0.265	0	0.01	0	0.305	0.55	0.635	0.055	0.375	0.115

* indicates the number of mosquitoes tested in each generation.

**Fig 4:**
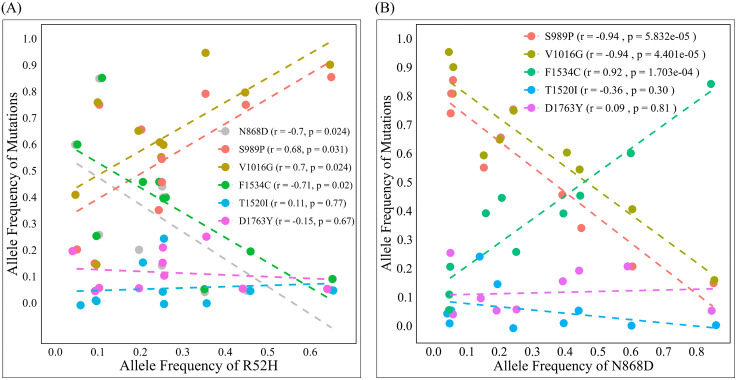
Correlation of allele frequencies of R52H (A) and N868D (B) with other *vgsc* mutations in field-collected *Aedes aegypti* in 2024. The correlation coefficient (r) and p-value (p) for each mutation are shown in parentheses. The linear regression line is represented by the dashed line.

The co-occurrence of R52H with other mutations is shown in Fig 4A. R52H exhibited a significantly positive correlation with S989P and V1016G and a significantly negative correlation with N868D and F1534C. We also observed that R52H occurred in the presence of S989P and V1016G but in the absence of F1534C, T1520I, and D1763Y in individual mosquitoes, although only a small number of mosquitoes with I/I (1) and Y/Y (3) were observed. We noticed that S989P and V1016G could occur without R52H (S7 Table). The co-occurrence of N868D with other mutations in the field population is shown in Fig 4B. A significant positive correlation was observed between N868D and F1534C, whereas significant negative correlations were identified between N868D and either S989P or V1016G. We also observed that N868D occurred in the presence of F1534C but in the absence of S989P, V1016G, and D1763Y in individual mosquitoes. Most mosquitoes that were homozygous for F1534C were homozygous for N868D (19/22) (S8 Table). We also observed a novel triple mutation, N868D+T1520I + F1534C, in one mosquito (S8 Table).

**Fig 5:**
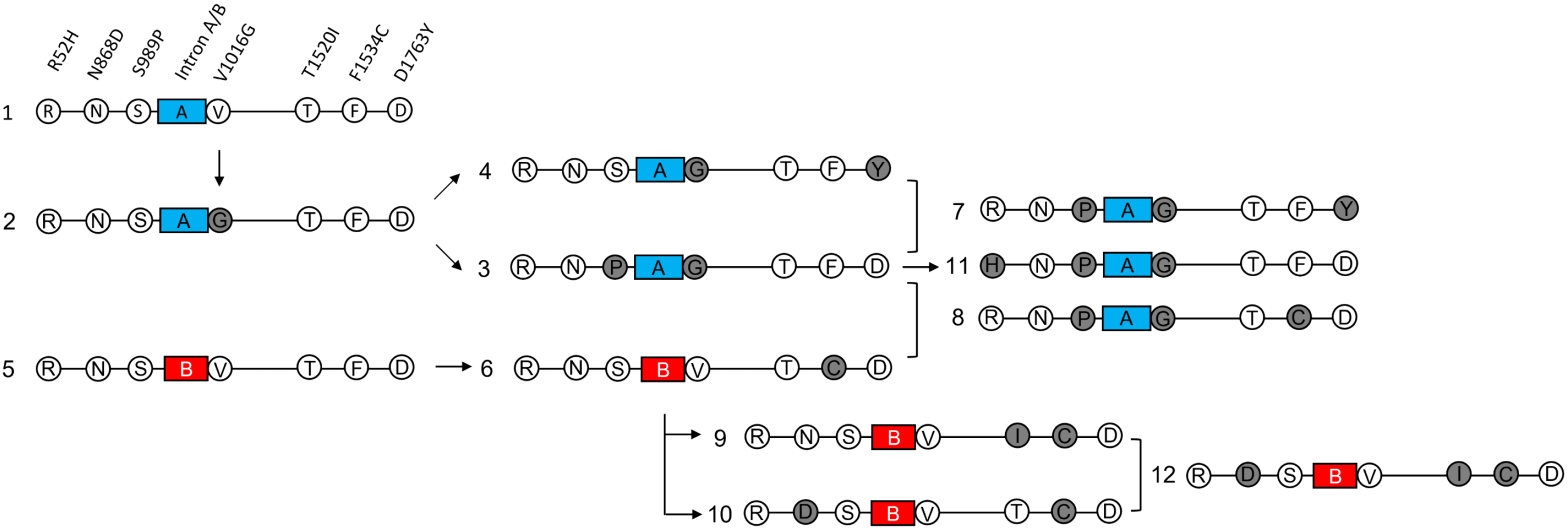
*vgsc* haplotypes and their sequential evolutionary events in *Aedes aegypti* in Taiwan. White and gray circles denote wild-type and mutant amino acids, respectively, whereas blue and red boxes indicate the group A and group B introns, respectively. The arrow indicates the evolution event of mutations. The right square bracket indicates the possible recombination of two existing haplotypes.

Based on our co-occurrence analysis and previous studies [19,20], we proposed 12 haplotypes and sequential evolutionary events in the *vgsc* of *Ae. aegypti* in Taiwan (Fig 5). We deduced that the specific unmutated haplotypes with two groups of introns were ancient haplotypes, which evolved into two lineages (Nos. 1 and 5). RNSaGTFD (No. 2, underlined is the mutant amino acid; a or b represent two groups of intron polymorphisms) was derived from RNSaVTFD (No. 1), and RNSaGTFD (No. 2) separated into two distinct lineages, namely RNPaGTFD (No. 3) and RNSaGTFY (No. 4). RNPaGTFD (No. 3) further evolved into HNPaGTFD (No. 11) or combined with RNSaGTFY (No. 4) to generate RNPaGTFY (No. 7). The specific unmutated haplotype with B introns, RNSbVTFD (No. 5), evolved into RNSbVTCD (No. 6). RNSbVICD (No. 9) and RDSbVTCD (No. 10), which were derived from RNSbVTCD (No. 6), further combined to generate RDSbVICD (No. 12). We also observed a combination of two lineages that produced RNPaGTCD (No. 8).

**Fig 6:**
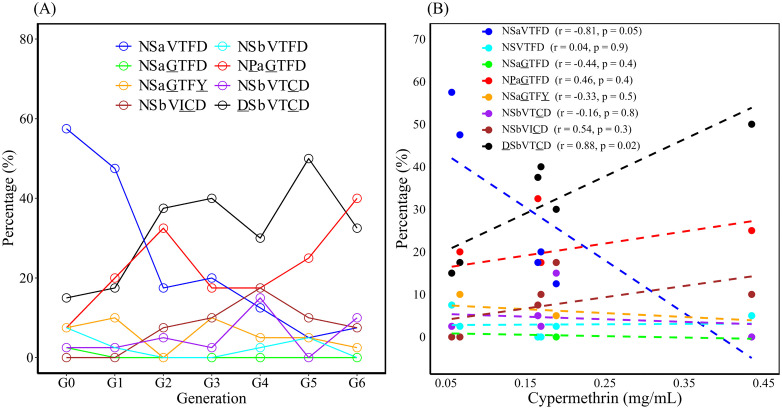
Variation of *vgsc* haplotypes (A) and the relationship between haplotype percentage and LC_50_ of cypermethrin (B) in sequential cypermethrin-selected *Aedes aegypti.* The correlation coefficient (r) and p-value (p) for each mutation are shown in parentheses. The linear regression line is represented by the dashed line.

The dynamic variation of haplotypes in G0–G6 is displayed in Fig 6A. An upward trend of DSbVTCD, with a peak in G5, was observed. NPaGTFD also exhibited an increasing trend, with two peaks in G2 and G6. NSaVTFD dramatically declined from 57.5% in G0 to 7.5% in G6. NSbVICD gradually increased in G0–G4 and subsequently decreased. A significantly positive correlation between DSbVTCD and LC_50_ of cypermethrin in G0–G5 and a significantly negative correlation between NSaVTFD and LC_50_ of cypermethrin in G0–G5 were observed (Fig 6B). However, no significant correlation was observed between LC_50_ and other haplotypes.

## Discussion

In this study, we proposed two novel pyrethroid resistance-associated mutations, R52H and N868D, by investigating cypermethrin-selected *Ae. aegypti* strain and genotyping the field population of *Ae. aegypti*. These two mutations occurred exclusively with previously reported resistance-conferring mutations, S989P+V1016G and F1534C. The general detection of R52H and N868D and their co-circulation with other resistance-associated mutations in the current field environment suggests that both mutations were preserved under high selection pressure. Overall, 12 *vgsc* haplotypes (seven mutations and two intron polymorphisms) with sequential evolutionary events were reported in this study, including N868D+T1520I+F1534C triple mutation, which was detected for the first time in *Ae. aegypti*.

Owing to next-generation WGS technology, novel *vgsc* mutations associated with pyrethroid resistance have been discovered efficiently [11,24,30,31]. In the present study, five *vgsc* mutations (R52H, D126N, P162H, A532E, and N868D) were identified as potential resistance-associated mutations. However, when we genotyped the mosquitoes of the G0, G3, and G6 populations individually, D126N, P162H, and A532E showed low allele frequencies (< 0.1), although the allele frequencies of D126N and P162H increased in later generations. Furthermore, D126N and A532E were absent, and the frequency of P162H was extremely low (0.01) in the field population collected in 2024. We detected two individuals harboring homozygous A532E in the G0 samples; however, the alleles in S989P, V1016G, T1520I, F1534C, and D1763Y were wild types. Previous studies have also not revealed the role of D126N, P162H, and A532E in *Ae. aegypti* resistance [41]. These results imply that D126N, P162H, and A532E revealed in WGS are either resistance-unrelated polymorphisms or are generated under laboratory-selection conditions.

A long-term surveillance study conducted in Taiwan between 2016 and 2023 observed the disappearance of wild-type mosquitoes (70.1% in 2016 to 0% in 2020–2023) and that three well-known resistance-related *vgsc* haplotypes, PGTFD, SVTCD, and SVICD, have emerged and become predominant (20.7% in 2016 to 92% in 2023) in the field population in Taiwan [20]. This suggests that the resistance of the current field *Ae. aegypti* has become more serious than in 2016. In cypermethrin-resistant mosquitoes, we observed that the frequency of N868D and the percentage of N868D+F1534C were significantly correlated with resistance levels. Moreover, the frequency of N868D in the field population in 2024 was 0.305. These results demonstrate that N868D is preserved for an extended period and co-circulates with other resistance-related *vgsc* mutations in the field population under selection pressure. A similar result was observed for R52H. The frequency of R52H was 0.265 in the field population in 2024, which was higher than that of the previously reported resistance-associated mutations, T1520I (0.055) and D1763Y (0.115) [17,42]. Although we could not correlate allele frequency of R52H and percentage of R52H+S989P+V1016G with the LC_50_ of cypermethrin owing to its low presence during cypermethrin selection, we observed a remarkable increase in R52H frequency in G6 compared with the frequency in G0 and G3 (Table 2). Moreover, the allele frequency of R52H increased noticeably in the field population in 2024 compared with that in 2016. This pattern suggests that R52H was amplified, maintained over time, and co-circulated with resistance-related *vgsc* mutations, implying its role in resistance. To the best of our knowledge, this is the first study to reveal the association of N868D and R52H with pyrethroid resistance in *Ae. aegypti*. Using the homology model of *Drosophila melanogaster* (P35500) in SWISS-MODEL, R52H aligns to the N-terminal cytoplasmic region, and N868D aligns to a residue in the N-terminal region of segment 3 in domain II [43]. Both positions are distinct from the canonical pyrethroid binding pocket, the pyrethroid receptor sites PyR1 and PyR2, in segments 5–6 of domains II and III [10,30]. This suggests that both mutations are unlikely to directly affect pyrethroid binding affinity, but may instead act through allosteric effects on channel gating or conformational dynamics. In addition, we cannot exclude the possibility that these mutations modulate or compensate for fitness costs associated with other *vgsc* mutations [44,45]. Further functional validation will be required to distinguish between these mechanisms.

Effective vector control is a means to interrupt dengue virus transmission that can reduce severe and fatal cases [46]. However, in *Ae. aegypti*, the F1534C mutation has achieved widespread global distribution, while the S989P+V1016G double mutation is predominantly found in Asian populations [47,48]. These pyrethroid resistance mutations seriously undermine vector control programs targeting *Aedes*-transmitted diseases [49]. Resistance phenotyping demonstrates that F1534C confers moderate resistance (3.6-fold and 12-fold to permethrin and deltamethrin, respectively), whereas S989P+V1016G produces more severe resistance phenotypes (48-fold and 33-fold to the same compounds, respectively) [30]. Moreover, the co-occurrence of multiple *vgsc* mutations has been proven to contribute to higher resistance than mutations that occur alone. Both F1534C and S989P+V1016G have been shown to provide resistance [10,50]. Previous studies have shown that neither T1520I nor V1016I alone confers resistance; however, each of these two mutations enhances F1534C-conferring resistance [42]. In addition, the concordance of S989P+V1016G and F1534C exhibited higher resistance than each of them. Also, L982W+F1534C presents extremely high resistance [30,50]. In this study, we found that N868D and R52H were strongly associated with F1534C and S989P+V1016G, respectively. In addition, the F1534C mutation co-occurred with N868D in 91% [(2*1)+(19*2)/(1 + 2 + 19)*2 in S8 Table)] of cases, and the S989P+V1016G mutation (noting that all S989P mutations were linked with V1016G) co-occurred with R52H in 59% [(16*1)+(14*2)/(7 + 16 + 14)*2 in S7 Table)] of cases in the current field population. These findings suggest that the N868D+F1534C and R52H+S989P+V1016G mutation combinations confer greater survival advantages compared to the previously reported F1534C and S989P+V1016G mutations under the heightened selection pressure of the current environment. Therefore, we strongly recommend that public health authorities implement continuous surveillance of the temporal and spatial dynamics of R52H and N868D mutations to inform appropriate vector control strategies. Furthermore, accumulating evidence indicates that insecticide-resistant *Ae. aegypti* exhibit altered vector competence that may influence disease transmission capacity [51–53]. We advocate for comprehensive studies investigating the vector competence of *Ae. aegypti* populations carrying R52H or N868D mutations to enable more accurate disease risk assessments.

In this study, the correlation between resistance level and haplotype percentage in G0–G5 cypermethrin-selected *Ae. aegypti* was analyzed. We found that DSbVTCD was significantly positively associated with resistance, and NSaVTFD was significantly negatively associated with resistance. We did not observe the association between resistance level and percentage of NPaGTFD, which is a well-proven resistance-conferring mutation [10,19,50]. Indeed, we observed an upward trend of this haplotype when selection pressure increased (Fig 6A), suggesting its role in resistance. We speculate that the absence of a significant difference is because of the “double peak” variation (G2 and G6) during selection (Fig 6A). Previous studies have suggested the fitness cost observed in mosquitoes with PG mutation [45]. We speculate that the “double peak” shape variation of NPaGTFD between G2 and G6 probably resulted from the balance between resistance and fitness cost. In addition, F1534C has been reported to exhibit resistance [10,50,54]. In this study, we observed that the proportion of DSbVTCD increased more rapidly than that of NSbVTCD during the selection process, indicating its potential to respond to insecticides. Our field data of 2024 showed a strongly concordant occurrence of N868D and F1534C, implying that N868D+F1534C is more suitable for survival in field environments than F1534C under selection pressure. Furthermore, previous studies have shown that S989P+V1016G is more potent in conferring resistance than F1534C [50,55]. In this study, an increasing trend of NPaGTFD with a decreasing trend of DSbVTCD was observed between G5 and G6. This observation suggests that NPaGTFD exhibits higher resistance than DSbVTCD. Because we only selected mosquitoes up to G6, additional studies should be conducted to validate our findings and draw conclusions. Furthermore, we observed a slight increase of NSbVICD in G0–G4, suggesting that this haplotype plays an important role in resistance; this finding is consistent with that of previous studies [42,56]. However, the three-generation increasing trend of NSbVICD was reversed when NPaGTFD increased after G4. This variation suggests that NSbVICD is more suitable for survival in the early stage of selection and exhibits less resistance than does NPaGTFD. Additionally, we detected the N868D+T1520I+F1534C haplotype in *Ae. aegypti* for the first time. As this haplotype was identified only once in our 2024 surveillance, further monitoring is warranted to track its prevalence and potential impact.

One limitation of the current study is the limited number of insecticide concentrations used in the adult bioassays. While the three concentrations provided a clear dose-response trend sufficient (*r* = 0.88, *p* < 0.019) for monitoring the selection progress across generations, the resulting LC_50_ values should be interpreted as estimates. Future studies aimed at precise toxicological characterization should employ a broader range of 5–6 concentrations covering 10–90% mortality to provide a more refined Probit model [32,57]. Nonetheless, the consistent methodology used across all tested generations allows for a reliable comparison of the relative increase in insecticide resistance. Furthermore, the LC_50_ values for G1–G5 were calculated relative to the G0 strain rather than a standard susceptible strain, specifically reflecting the dynamics of resistance development under selection. Consequently, the absolute resistance levels compared to a fully susceptible population remain to be determined in future studies.

In conclusion, technological advancements have enhanced the detection of novel resistance-associated mutations. In this study, we identified two new *vgsc* mutations, R52H and N868D, linked to *Ae. aegypti* resistance. Our findings suggest the ongoing emergence of resistance mutations, highlighting the increasing challenges in disease vector control in Taiwan and worldwide. Understanding the functional impact of these mutations is essential for comprehensive assessment of resistance levels and vector competence, thereby informing evidence-based policy decisions in vector management programs and disease risk evaluation. This study offers valuable insights for future research and highlights the importance of continuous resistance monitoring to enhance vector and vector-borne disease control strategies.

## Supporting information

S1 Fig Sequencing chromatograms showing the nucleotide changes underlying the N868D (A) and R52H (B) amino acid substitutions. For N868D, NN, ND, and DD represent the wild-type, heterozygous, and homozygous genotypes, respectively; similarly, RR, RH, and HH represent the corresponding genotypes for R52H. Nucleotide sequences are shown before the colon, and the corresponding amino acid states are shown after the colon.
(PPTX)

S1 Table The LC_50_ of cypermethrin of G0–G6 *Ae. aegypti* female. Mosquitoes were treated with three concentrations of cypermethrin in each generation, and the knockdown rate, mortality, and LC_50_ were calculated.
(XLSX)

S2 Table Polymerase chain reaction and sequencing primers used in this study. (XLSX)

S3 Table Knockdown rate and mortality of G0–G5 female and male *Aedes aegypti* selected according to LC_50_ of cypermethrin. (XLSX)

S4 Table Summary of whole-genome sequencing of G0, G3, and G6 *Aedes aegypti* strain in this study. (XLSX)

S5 Table Mutation frequencies of N868D, S989P, V1016G, F1534C, T1520I, and D1763Y in G0–G6 *Aedes aegypti.* (XLSX)

S6 Table Mutation frequencies of R52H, D126N, P162H, and A532E in live and dead field-collected *Aedes aegypti* after cypermethrin exposure. w and m denote the wild-type and mutant allele of each mutation. (XLSX)

S7 Table Co-occurrence of R52H with other mutations in *Aedes aegypti* in 2024. (XLSX)

S8 Table Co-occurrence of N868D with other mutations in *Aedes aegypti* in 2024. (XLSX)
