# Supplementary material for: Discovery of two novel mutations, R52H and N868D, in the voltage-gated sodium channel of Aedes aegypti associated with pyrethroid resistance
Source: PLoS Negl Trop Dis. 2026 May 26;20(5):e0014393. doi: 10.1371/journal.pntd.0014393 (PMC13225630; doi:10.1371/journal.pntd.0014393)
Supplement: S1 Fig — For N868D, NN, ND, and DD represent the wild-type, heterozygous, and homozygous genotypes, respectively; similarly, RR, RH, and HH represent the corresponding genotypes for R52H. Nucleotide sequences are shown before the colon, and the corresponding amino acid states are shown after the colon. (PPTX) [file pntd.0014393.s001.pptx]

## Slide 1
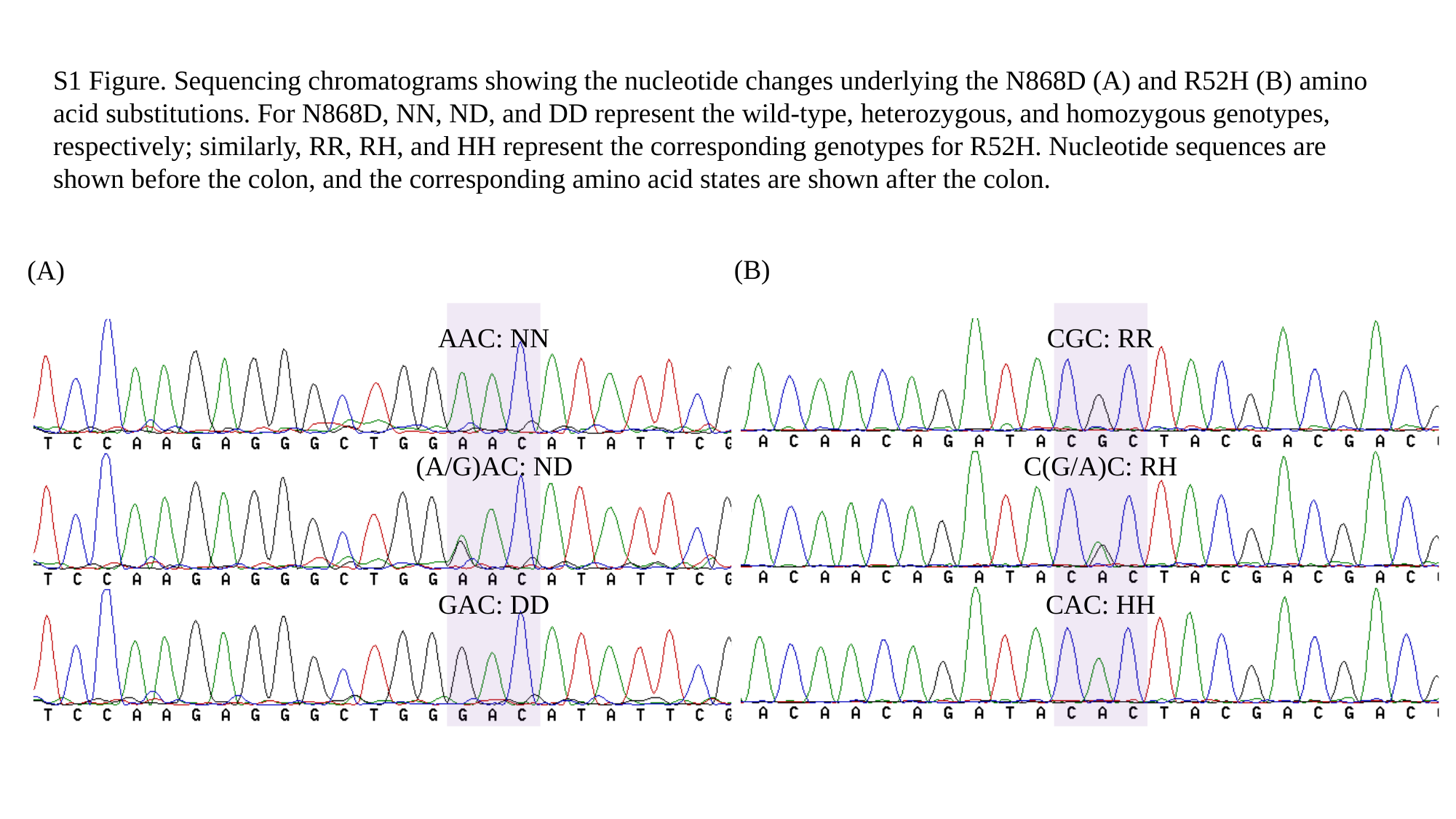

S1 Figure. Sequencing chromatograms showing the nucleotide changes underlying the N868D (A) and R52H (B) amino acid substitutions. For N868D, NN, ND, and DD represent the wild-type, heterozygous, and homozygous genotypes, respectively; similarly, RR, RH, and HH represent the corresponding genotypes for R52H. Nucleotide sequences are shown before the colon, and the corresponding amino acid states are shown after the colon.
(B)
(A)
CGC: RR
C(G/A)C: RH
CAC: HH
AAC: NN
(A/G)AC: ND
GAC: DD
